# 

**Published:** 1986-08

**Authors:** 


**Editor**
Mr M. G. Wilson
*40 Coombe Lane, Bristol BS9 2BJ*
*Tel: 0272-682320*

**Assistant Editor**
Dr P. R. Goddard

**Secretary Editorial Committee**
Mr B. P. Jones,
*Medical Librarian, Bristol University*

**Correspondents**
Dr A. E. Adam
Dr J. D. Davies
Dr J. Jancar
Dr H. G. Mather
Professor G. M. Stirratt
Dr J. L. G. Thomson
Dr M. J. Whitfield
Professor G. K. Wilcock
Professor R. C. N. Williamson
Dr D. W. Wright

**BRISTOL MEDICO-CHIRURGICAL SOCIETY**

**President**
Dr J. Jancar

**Secretary**
Mr R. N. Baird,
*23 Old Sneed Park, Bristol BS9 1RG*

**Treasurer**
Dr N. C. Tricks,
*The Mill, Wrington, Bristol*

**Published by**
**Hermiston Publications,**
*BMA Scottish House,*
*7 Drumsheugh Gardens,*
*Edinburgh EH3 7QP*

**Production Manager**
June Macnab

**Typesetting by**
*Avon Communications Ltd., The Sion,*
*Crown Glass Place, Nailsea, Bristol BS19 2EP*

**Printed by**
*Scottish County Press, Sherwood Industrial Estate,*
*Bonnyrigg, Midlothian*

### BRISTOL MEDICINE

Established 1883

### BRISTOL MEDICO-CHIRURGICAL JOURNAL

**AUGUST 1986**
**Volume 101 (iv)**

Published bi-monthly and distributed free of charge to all doctors in the South West of England. Overseas subscription £12 per annum.

Scire est nescire, nisi id me alius sciret.
*Lucilius (180–102 BC)*

**Bristol Medicine acknowledges with gratitude the support and encouragement it receives from Bristol-Myers Pharmaceuticals**

